# Sexual violence amongst rural women living with HIV: prevalence, forms and lived experiences at health facilities in southwestern Uganda

**DOI:** 10.11604/pamj-oh.2022.8.18.35525

**Published:** 2022-07-27

**Authors:** Gerald Mwebembezi, Beneth Kaginda Tusiime, Ebenezer Felex Sikon, Nabatanzi Esther Sanyu, Kenneth Agaba, Scarlet Josephine Nakaweesa, Godfrey Zari Rukundo

**Affiliations:** 1 Mbarara University of Science and Technology, Faculty of Medicine, P. O. Box 1410, Mbarara City, Uganda

**Keywords:** HIV/AIDS, intimate partner violence, sexual violence, experiences, Southwestern Uganda

## Abstract

**Introduction:**

sexual violence exists in different forms and contexts. It has different consequences including HIV/AIDS. There is limited information on the adoption of services to the victims. In this study, we investigated the prevalence, forms and personal experiences of sexual violence amongst women living with HIV/AIDS in rural southwestern Uganda.

**Methods:**

we conducted a cross-sectional survey among 324 women living with HIV. We also conducted 11 in-depth interviews among women living with HIV in the same area. We collected data on socio-demographics, participants’ experiences, forms of sexual violence, and the services for the victims. The in-depth interviews were conducted using a semi-structured interview guide. The quantitative data were analyzed at 95% confidence interval using Chi-square and logistic regression. Transcripts were cleaned were independently coded and any disagreements encountered during in the coding were resolved at each step. During the coding process, duplicates were removed. Similar quotes were grouped together under broad themes while ensuring inclusion of data from different sources. We used thematic content analysis to generate themes and sub-themes.

**Results:**

the mean age of the participants was 35.05 ± 12.21 years. Of the 324 women that participated in the study 68.8% were married 67.2% had primary level of education and were peasant farmers (70.1%). Sexual violence was experienced by 32.7% of the participants and this included sexual humiliation, forced genital touching and insertion of objects. Shame was the most commonly lived experience and sexual partners were the most common perpetrators of sexual violence. According to our findings, marriage (OR = 0.0250, 95%CI 0.069–0.905, p = 0.035) was a protective factor.

**Conclusion:**

sexual violence is commonly experienced by women living with HIV and attending health facilities in rural southwestern Uganda. Fear of shame negatively affects disclosure by the victims. Screening for sexual violence could be helpful in exposing the vice if it is integrated in routine HIV care for women.

## Introduction

Violence is a term that exists in different cultures and languages [1]. However, it may take different forms and it may be perceived differently in various settings. Overall, violence is the use of force to destroy, injure, damage or abuse [2]. The violence can be physical, emotional, sexual or economic. It is possible for an individual to suffer all the forms of violence at the same period. Generally, women and girls suffer more violence than men especially sexual violence [3]. The impact of violence can be long lasting. According to previous studies, about one third of women are suffer from sexual violence especially those who live in the developing world [4]. Although sexual violence has many definitions, it can broadly be as any sexual act, attempt to obtain a sexual act, unwanted sexual comments or advances, or acts to traffic, or otherwise directed, against a person’s sexuality using coercion, by any other person regardless of their relationship to the victim’ [5]. In sub-Saharan Africa, approximately 19% of women suffer sexual violence [6] but at least 22% of Ugandan women in a reproductive age (15–49 years) suffer sexual violence [7–9]. Although HIV/AIDS is an outcome of sexual violence it is also a risk factor [10,11]. When sexual violence happens amongst women with HIV/AIDS, it can further lead to reduced adherence to antiretroviral therapy (ART) [12] which is their most hope for survival and wellbeing [13]. It is not clear if the sufferers of sexual violence are able to access services.

Sexual violence exists in different contexts and forms (compelled marriage, rape, undesirable sexual advances, sexual harassment, sexual abuse of children, forced abortion and compelled prostitution and denial of the right to apply birth control) [14]. It has negative consequences on the welfare of the sufferers and their families. In addition to the physical and social consequences, sexual violence has a psychological impact including post-traumatic stress disorder, depression, suicidal thoughts, substance abuse and obsessive-compulsive disorder [13,15]. Although they may no longer want to discuss the occasion itself, sufferers of sexual violence might seek medical help [16,17]. The medical professionals in primary health care are crucial in identifying and responding to the needs of the victims of sexual violence [18]. However, this form of violence may go undetected. This calls for sensitization and capacity building of the medical professionals who are the gatekeepers of health care. This study investigated prevalence, forms and personal experiences of sexual violence amongst women living with HIV/AIDS at selected health centers in rural southwestern Uganda.

## Methods

### Study design:

this was a cross sectional study in March and April, 2021. We collected data on socio-demographics, participants’ experiences, forms of sexual violence, services sought following sexual violence encounter and screening for sexual violence by health workers. We conducted in-depth interviews using a semi-structured interview guide.

### Study setting and population:

this study was conducted at three health facilities in the district of Buhweju. The district is rural and about 71Km from Mbarara city. The district has a population of about 144,100 people of whom 51.1% are women [19]. The district has 2994 people living with HIV and 60% of them are women. The district has no general hospital but offers free HIV/AIDS services at a one health center IV and three health center IIIs. The participants in this study were women living with HIV 15 years or older. Women who were mentally unstable were excluded. They would not have been able to provide informed consent.

### Study variables:

prevalence of sexual violence was the main outcome variable. It was defined as any sexual act, attempt to obtain a sexual act, unwanted sexual comments or advances, or acts to traffic, or otherwise directed, against a person’s sexuality using coercion, by any other person regardless of their relationship to the victim’. The secondary outcomes were forms and the women’s experiences of sexual violence. The independent variables were: age, education, level of income, marital status, distance to the nearest health facility and occupation.

### Data collection tools:

we used the Norvold Abuse Questionnaire (NorAQ) [20] to collect the quantitative data. The NorAQ was researcher administered to ensure consistency since some of the participants were not able to read and write. An interview guide was used to collect qualitative information on individuals’ experiences of sexual violence. The interview guide had open questions on the understanding of sexual violence, personal experiences of sexual violence, services sought and accessibility of services. Some of the interview questions were: What do you know about sexual violence? What are the examples of sexual violence? If it is okay with you, please tell us about your experience of sexual violence (with probes for personal experiences, perpetrators and what was done to them). How did you cope after the attack? What health services did you seek? (If not; why? Probe for HIV testing, emergency contraception and counselling). Who provides these services? How accessible are the services?

### Data collection:

using consecutive sampling, we selected participants for the survey on weekdays when they attended the HIV clinics (Tuesday, Wednesday and Thursday). Potential participants were first approached and given study related information. Those that signed the consent forms were allowed to participate. Some of the participants who were found to have experienced sexual violence were purposively selected and requested to consent for in-depth interviews. The interviews were conducted by trained research assistants face-to-face, in Runyankore-Rukiga, the locally spoken language. After conducting 11 in-depth interviews, no more new information emerged.

### Sample size:

we used Cochran’s formula to estimate the required sample size for quantitative data:

$$n=\frac{Z^{2}pq}{e^{2}}$$

(Where Z = 1.96, p = 0.6, q = 0.4, e = 0.05). The sample size for the qualitative interviews was determined by the principle of data saturation.

### Data management and analysis:

we analyzed the quantitative data using SPSS version 23. We used Chi-square for categorical variables, and logistic regression to assess associations at 95% level of confidence at p≥0.05. For the qualitative data, the audio interviews were transcribed verbatim, anonymized and translated into English by a translation expert. Transcripts were cleaned, and independently coded. During the coding process, duplicates were removed. Similar quotes were grouped together under broad themes and ensuring inclusion of data from different sources. We used thematic content analysis [21] to generate themes and subthemes.

### Ethical considerations:

ethical approval was obtained from the Research Ethical Committee of Mbarara University of Science and Technology (08/12–20). We also obtained administrative clearance from the District Health Officer. Informed consent was obtained from all study participants provided before participating in the study and participation was voluntary. Data collected from study participants did not contain identifies like names, phone numbers and initials to ensure anonymity. The data was kept confidential and only accessed by the research team. During data collection, private rooms were used in which only the research assistant and the interviewer were present to ensure confidentiality. The research assistants had no prior relationship with any of the participants before the interviews.

## Results

### Sociodemographic information:

the mean age of the 324 study participants was 35.05 ± 12.2 12 years with the age range of 18–76 years and median of 32 years (Table 1). Most of them were married (68.8%), with primary level of education (67.2%) and were peasant farmers (70.1%).

### Descriptive analysis:

about one third of the women 32.7% (106/324) experienced sexual violence especially among the divorced (40.0%), older women (43.8%) and those with no formal education (36.4%) as seen in Table 1. Sexual violence (mainly sexual humiliation, forced genital touching and insertion of objects into genitalia) was mainly perpetrated by the victims’ sexual partners especially husbands (Table 2).

### Bivariate analysis:

apart from marriage which was protective against sexual violence (p = 0.035, 95% CI 0.069–0.905), other study variables were not statistically significant (Table 3) and we did not do multivariable analysis.

## Qualitative data

### Forms of sexual violence:

during in-depth interviews, participants were asked about the forms of sexual violence. They reported different forms of sexual violence but the most common forms were sexual humiliation, insertion of an object into genitalia, rape and forced sexual intercourse in menstruation periods and forced genital touching as described in the quote below. “*He ambushes you, grabs you by force and then tells you a lot of scaring things, like this so-called friend of mine…. he told me how much he wants to have sex with me. You also tell him the truth, how you cannot manage such things and you do not want anything to do with that. Remember us as women we do not have that much energy that men have, so when the man handles you, you will not defend yourself and eventually he will over power you, then force you into sex.” IDI_7, 02/04/2021*. The victims of sexual violence seem to perceive themselves as weak and taken advantage of by the perpetrators of violence who on the other hand are seen as forceful and strong.

### Sexual violence perpetrators:

women are at risk of being violated by many types of people especially their sexual partners. Under normal circumstances, partners would be supportive and protective. “*…actually, at one time he almost cut off my head; …..just check here below my eye there is a scar… we went to sleep that night then he suddenly woke me up. ….. I thought he was joking, he was demanding for sex, he became so rough with me and almost cut my head off just because he wanted sex.” IDI_07, 31/03/2021*.

### Seeking health services after sexual violence:

the health services to victims are generally available in the health but some victims don’t seek them. Majority of the affected women did not disclose the incidents to other people and the majority (85%) did not seek medical help for the sexual violence. Majority did not receive the services probably due to fear of shame. The available services include HIV testing, emergency contraception, Post Exposure Prophylaxis (PEP) and psychosocial counseling. *“…assistance at the health facility seems to be available but we people from the communities are not aware that we can access help and be assisted but for sure the assistance is very available.” IDI_07, 31/03/2021*. Some of the participants sought the services late. They did not seek the services immediately as recommended. *“It was after about five days; after coming from the police station, they told me to go to the hospital…” IDI_09, 9/4/2021.“…I was sexually assaulted on a Friday at about 2:00pm; the following day was a Saturday which is a weekend, then a Sunday, so I decided to go on Tuesday for a healthy check.” IDI_07, 31/03/2021*. The late seeking of services could have affected the effectiveness of the interventions such as post-exposure prophylaxis.

### Disclosure of sexual violence:

victims of sexual violence coped in various ways such as telling someone about the incident (parents, friends and neighbors, counselors and nurses) and change of residence to avoid the perpetrator. However, the majority did not disclose their experiences: *“I didn’t tell them anything about the incident that happened; actually, the health worker is the one who asked me why I had come for a pregnancy test and I replied that I slept with someone I didn’t understand very well.” IDI_07, 31/03/2021*. The fact that they did not disclose prevented them from receiving the appropriate assessment and treatment. The lack of assurance and that disclosure would not have led to stigma affected the victims’ response to the sexual violence.

### Effects of sexual violence:

sexual violence had several negative effects on the victims including getting the HIV infection, unwanted pregnancies, unstable relationships, early marriages, death threats, experience of shame and physical injuries: *“After being sexually harassed I found out that I was HIV positive and my family members neglected me. So, I became desperate; I almost became crazy… IDI_06, 08/04/2021*.

## Discussion

Approximately one third of the women experienced sexual violence. It was mainly experienced in form of sexual humiliation, insertion of an object into genitalia and forced genital touching. The main perpetrators were their sexual partners and few victims disclosed the incidents and sought medical help. Our findings contribute to the body of knowledge that may be useful in provision of care to women living with HIV who are also experience sexual violence in developing countries such as Uganda. Having a prevalence of 32.7% among women living with HIV is unacceptable. This double burden (living with HIV and experiencing sexual violence) has long lasting consequences. Sexual violence is a sensitive topic that victims fear to talk about and may not receive the necessary help. Secondly, the combination of sexual violence and HIV makes the lives of the affected women more difficult. It may lead to poor quality of life, poor treatment adherence and poor treatment outcomes if not addressed. The prevalence similar to the global rate of 35.6% [4] but it is much higher than the national prevalence of 22.8% reported by the Uganda Bureau of Statistics [7]. It is also higher than 29% reported in a previous Ugandan study [9] and the studies in South Africa and Rwanda [22,23]. On the other hand, our prevalence is lower than what has been in the United States [24] possibly due to the fact that the study populations and settings are quite different.

According to previous studies there are different factors associated with sexual violence. However, in our study only being married was significantly protective against sexual violence. Some of the experiences may be considered as normal experiences in marriage. This finding should be taken with caution since there are contradicting reports in literature. There is need for more studies to assess the protective association between marriage and sexual violence in the Ugandan setting. Similar to previous studies, insertion of the penis or any other object into the vagina, rectum or mouth was the most common (34.3%) form of sexual violence [5]. In addition, the rural setting and the low socioeconomic status of the women in our study could have influenced their low bargaining power and low rates of disclosure. The forms of sexual violence in our study were also reported as the main forms experienced in studies done in DRC and South Africa [25,26]. Prevalence of sexual violence was higher among older divorced women with no formal education. It is possible that older women are not able to defend themselves against the perpetrators. In addition, they may be able to cope better with abuse if it has been going on for long. Leaving a marriage because of abuse may attract more criticism if the woman is older. On the other hand, women who are uneducated may not have the required knowledge to fight for their own rights and may therefore, be taken for granted by the male sexual partners. Lack of education is also associated with high levels of poverty and vulnerability. Although we did not determine whether divorce preceded sexual violence, it is possible that the high burden of sexual violence among the divorced women was the reason for divorce. Most of the affected women did not disclose the incidents to the health workers despite the fact that health care providers were the first professional providers the victims came into contact with. The few women that sought care did not do so immediately, despite service availability. Yet, the services are more effective within a window of 3–5 days after possible exposure. When services are sought late, the preventive strategies such as Post Exposure prophylaxis (PEP) and emergency contraception [18,26–28].

A South African study reported that being a victim of sexual violence is stigmatizing and shaming and women may fear to talk about it [29]. Similarly, a study conducted in Togo, West Africa, observed that only a 3^rd^ of the participants disclosed the real cause of injuries to the medical staff and had not been referred to local organizations for appropriate psychological help [30]. The WHO policy guidelines state that women who experience sexual violence seek health care more than non-abused women but they do not disclose the associated violence [18]. previous studies [31,32] have also indicated that sexual violence is associated with poor health seeking behaviour [33]. Similar findings have been reported in studies conducted in Swaziland, South Africa and Kenya [25,34,35]. Although much progress has been made in HIV/AIDS care, sexual violence has not received appropriate attention during routine HIV services [35]. Very few participants had been asked by health workers about sexual violence. Screening and responding to the needs of the victims of sexual violence can encourage women to always seek appropriate services [36]. In Uganda, there are laws, regulations, policies and government programmes against sexual violence but implementing them has continued to be a challenge.

### Strengths and limitations:

the study was conducted in a very remote and hard to reach area where little research has been conducted on sexual violence. We expect that the findings of this study will act as a reference for future scientific studies. The risk of COVID-19 travel and gathering restrictions were a limiting factor. We provided free face masks and hand sanitizers to prevent the spread COVID-19 and the research assistants went to the health facilities. If this study was conducted outside the COVID-19 pandemic, it is possible that the participants would have been slightly different.

## Conclusion

Sexual violence is a common experience among women seeking HIV care in rural southwestern Uganda. Most of the sexual violence is perpetrated by the victim’s sexual partners and the majority do not disclose the incidents.

## Figures and Tables

**Table 1:** prevalence of sexual violence and associated factors among 324 HIV women living with HIV at health facilities in Buhweju district, southwestern Uganda

Variable	Description of variables	Total (N=324)	No sexual violence n (%) [218 (67.3)]	Experienced sexual violence n (%) [106 (32.7)]
Age (years)	18–30	146 (45.1)	94 (66.7)	47 (33.3)
	31–59	162 (50.0)	115 (68.9)	52 (31.1)
	60+	16 (4.9)	9 (56.2)	7 (43.8)
Marital status	Single	28 (8.6)	24 (85.7)	4 (14.3)
	Married	223 (68.8)	147 (65.9)	76 (34.1)
	Widowed	43 (13.3)	29 (67.4)	14 (32.6)
	Divorced	30 (9.3)	18 (60.0)	12 (40.0)
	No formal education	11 (3.4)	7 (63.6)	4 (36.4)
Education	Primary	247 (67.2)	163 (66.0)	84 (34.0)
	Secondary	60 (18.5)	42 (70.0)	18 (30.0)
	Tertiary	6 (1.9)	6 (100)	0 (0.0)
Employment	Business/self-employed	48 (14.8)	29 (60.4)	19 (39.6)
	Employed	13 (4.0)	10 (76.9)	3 (23.1)
	Unemployed	36 (11.1)	22 (61.1)	14 (38.9)
	Farmer/peasant	227 (70.1)	157 (69.2)	70 (30.8)
Distance to health facility	1Km	38 (11.7)	25 (65.8)	13 (34.2)
	3Km	159 (49.1)	114 (71.7)	45 (28.3)
	5Km	99 (30.6)	62 (62.6)	37 (37.4)
	More than 5Km	28 (8.6)	17 (60.7)	11 (39.3)

**Table 2:** forms of sexual violence, perpetrators and supports sought among 324 women living with HIV at health facilities in Buhweju district, southwestern Uganda

Form of sexual violence	Frequency	Percentage
Sexual humiliation	85	26.2
Forced genital touching	149	46.0
Insertion of an object into genitalia	111	34.3
**Perpetrator of violence**		
Husband	165	50.9
Ex-husband	28	8.6
Boy friend	19	5.9
Others	28	8.6
Fear of perpetrator		
Fear of the perpetrator	132	40.7
**Disclosure of sexual violence**		
Disclosure to someone	132	22.1
**Person disclosed to**		
Current partner	24	7.4
Father	3	0.9
Friend	13	4.0
**HIV services**		
Sought medical help	49	15.1
Post-exposure prophylaxis	16	4.9
Received HIV test after sexual violence	30	9.3
Emergency contraceptives	18	5.6
**Provider of care/support**		
Own family	12	3.7
Friend	15	4.6
Health workers screen for sexual violence	155	47.8
**Frequency of sexual violence screening**		
Very often	108	33.3
Not often	134	41.4

**Table 3:** factors associated with sexual violence among 324 women living with HIV at health facilities in Buhweju district, southwestern Uganda

Variable	Description	Unadjusted OR	95% CI	P-value
**Age (years)**	18–30	1		
	31–59	0.643	0.225–1.833	0.409
	60+	0.581	0.205–1.646	0.307
	Single	1		
**Marital status**	Married	0.250	0.069–0.905	0.035
	Widowed	0.776	0.355–1.694	0.524
	Divorced	0.724	0.275–1.910	0.514
**Education**	No formal education	1		
	Primary	1.52	0.34–5.84	0.539
	Secondary and tertiary	1.37	0.75–2.51	0.301
**Employment**	Business/self-employed	1		
	Employed	1.469	0.772–2.796	0.241
	Unemployed	0.673	0.180–2.520	0.556
	Farmer/peasant	1.427	0.690–2.952	0.337
**Distance to health facility**	1Km	1		
	3Km	0.804	0.292–2.21	0.672
	5Km	0.610	0.265–1.40	0.245
	More than 5Km	0.922	0.390–2.18	0.854

## What is known about this topic

- Sexual violence is more common among women than men living with HIV.

## What this study adds

- This study contributes to the current literature about the prevalence of sexual violence among women living with HIV in Uganda;
- It also highlights the different forms of sexual violence suffered by women in rural Uganda;
- It also reveals that fear of breaking confidentiality and the shame associated with disclosing sexual violence are among the barriers why women do not seek for help when they are sexually violated.
